# Retraction Note to: Mechanistic attributes of S100A7 (psoriasin) in resistance of anoikis resulting tumor progression in squamous cell carcinoma of the oral cavity

**DOI:** 10.1186/s12935-021-01786-2

**Published:** 2021-02-01

**Authors:** Kaushik Kumar Dey, Siddik Sarkar, Ipsita Pal, Subhasis Das, Goutam Dey, Rashmi Bharti, Payel Banik, Joygopal Roy, Sukumar Maity, Indranil Kulavi, Mahitosh Mandal

**Affiliations:** 1 School of Medical Science and Technology, Indian Institute of Technology, Kharagpur, West Bengal 721302 India; 2 Dr Rafi Ahmed Dental College and Hospital, Kolkata, West Bengal 700014 India; 3 Calcutta Medical College, Kolkata, West Bengal 700073 India; 4 Bankura Sammilani Medical College, Bankura, West Bengal 722101 India

## Retraction to: Cancer Cell Int (2015) 15:74 10.1186/s12935-015-0226-9

The authors have retracted this article [1] because there are anomalies in the data shown in Figures 3, 4, and 5.

All authors agree with this retraction. The authors are repeating their study and will submit a new manuscript for peer review.
